# Regional Distribution Shifts Help Explain Local Changes in Wintering Raptor Abundance: Implications for Interpreting Population Trends

**DOI:** 10.1371/journal.pone.0086814

**Published:** 2014-01-22

**Authors:** Neil Paprocki, Julie A. Heath, Stephen J. Novak

**Affiliations:** 1 Department of Biological Sciences, Boise State University, Boise, Idaho, United States of America; 2 Raptor Research Center, Boise State University, Boise, Idaho, United States of America; Norwegian Polar Institute, Norway

## Abstract

Studies of multiple taxa across broad-scales suggest that species distributions are shifting poleward in response to global climate change. Recognizing the influence of distribution shifts on population indices will be an important part of interpreting trends within management units because current practice often assumes that changes in local populations reflect local habitat conditions. However, the individual- and population-level processes that drive distribution shifts may occur across a large, regional scale and have little to do with the habitats within the management unit. We examined the latitudinal center of abundance for the winter distributions of six western North America raptor species using Christmas Bird Counts from 1975–2011. Also, we considered whether population indices within western North America Bird Conservation Regions (BCRs) were explained by distribution shifts. All six raptors had significant poleward shifts in their wintering distributions over time. Rough-legged Hawks (*Buteo lagopus*) and Golden Eagles (*Aquila chrysaetos*) showed the fastest rate of change, with 8.41 km yr^−1^ and 7.74 km yr^−1^ shifts, respectively. Raptors may be particularly responsive to warming winters because of variable migration tendencies, intraspecific competition for nesting sites that drives males to winter farther north, or both. Overall, 40% of BCR population trend models were improved by incorporating information about wintering distributions; however, support for the effect of distribution on BCR indices varied by species with Rough-legged Hawks showing the most evidence. These results emphasize the importance of understanding how regional distribution shifts influence local-scale population indices. If global climate change is altering distribution patterns, then trends within some management units may not reflect changes in local habitat conditions. The methods used to monitor and manage bird populations within local BCRs will fundamentally change as species experience changes in distribution in response to climate change.

## Introduction

Animal distribution shifts in relation to global climate change have been well documented [1]–[3]; with many comparative studies focusing on a large number of taxa across broad geographic areas [4]–[6]. As distributions shift, long-term monitoring projects will likely detect changes in local population indices. Studies of wintering birds have found variation in population trends at the Bird Conservation Region (BCR) level [7], [8]. Link et al. [7] found American Black Duck (*Anas rupripes*) populations increased in northern BCRs and declined in central and southern BCRs, while overall regional population indices remained stable. These local population trend differences may be difficult to interpret, but could be partly explained by distribution shifts. Bart et al. [9] examined population trends of wintering North American shorebirds and concluded that population declines in wintering shorebirds were most likely a result of declining breeding populations, but they could not rule out the possibility that shorebird distribution shifts were responsible for observed declines.

The mechanisms that drive distribution shifts in response to climate change do not necessarily indicate changing land uses that lead to decreased habitat suitability in southern areas or increased habitat suitability in northern areas. For example, warmer temperatures and increased climate suitability may allow birds to winter closer to, or stay on, nesting grounds. Warmer winters and shorter migration distances may have carry-over effects on breeding birds [10], such as early arrival at nesting sites allowing individuals to secure higher quality territories [11]–[13]. In addition, distribution shifts may depend on population- or species-specific life histories [14]–[16], geography [17], [18], or regional climate-change patterns [11]. Assessment of distribution shifts on a scale that is biologically relevant to a population may allow for better estimates of how and why populations change over time.

Raptors may be particularly responsive to warming winters because many species have highly variable migration tendencies [19], or migration distances [20]. In addition, high intraspecific competition for quality nest sites that drives protandry (earlier male arrival to breeding areas than female) may facilitate poleward distribution shifts as climate change proceeds [21], [22]. Raptor distribution shifts may vary regionally, in comparison to continent-wide estimates, because many species have strong north-south patterns of migratory connectivity [23] and weaker east-west population connectivity. Individual species of raptors may also vary in their response to climate change patterns given that projected changes are greatest at higher latitudes. Several raptor species breed exclusively at arctic latitudes [24], while the range of other species encompasses arctic and temperate regions [25], or only temperate regions [26]. Finally, climate change patterns vary with distance from the coast [27], and this may also correspond to regional differences in distribution responses given the widespread occurrence of most raptor species.

Our first objective was to use data from the National Audubon Society’s Christmas Bird Count (CBC) [28] to investigate latitudinal shifts in winter distributions of western North America raptors over the past 36 years (i.e., a regional distribution shift). We predicted that raptors would show northward shifts in their latitudinal center of abundance over time and that estimates of distribution shifts for western raptors would differ from continent-wide estimates. Secondly, we examined whether regional latitudinal center of abundance helped explain local CBC population indices within western Bird Conservation Regions. Several raptor species are monitored during non-breeding surveys, like the CBC, because it is difficult to adequately sample breeding populations [29]. We used an information-theoretic framework to compare support for models containing a combination of the following predictors: 1) “residual latitude center of abundance” models that indicate how regional distribution shifts contribute to local BCR population trends independent of a year effect, 2) “year” models that represent change over time, and 3) “intercept-only” models that represent no explainable change in BCR populations. We focused on six raptor species that are common in western North America, highly detectable in surveys, and whose wintering distribution is well sampled by Christmas Bird Counts: American Kestrels (*Falco sparverius*), Golden Eagles (*Aquila chrysaetos*), Northern Harriers (*Circus cyaneus*), Prairie Falcons (*Falco mexicanus*), Red-tailed Hawks (*Buteo jamaicensis*), and Rough-legged Hawks (*Buteo lagopus*).

## Methods

### Latitudinal Center of Abundance

Christmas Bird Counts were conducted by National Audubon Society volunteers who counted all birds detected within a designated survey area of one 24-km diameter circle, on 1 day between 14 December and 5 January [28]. Each yearly CBC was conducted by a number of observers that varied through time and among circles. Effort at a circle was reported as the number of observers multiplied by the number of survey hours. We used CBC data from 1975 to 2011 because reporting of observer effort became relatively consistent after 1975 [5]. We modified La Sorte and Thompson’s [5] approach of selecting long-term circles sampled at least once during a minimum of 9 of 12, 3-year time periods (e.g., 1975–1977, 1978–1980, …, 2008–2011; Table 1) to ensure adequate sampling over our study period. The 2008–2011 time period contained four survey years. We removed CBC circles where a given species was never encountered. We also removed circle data for years when observer effort was missing or recorded as zero (n = 319) [30]. To eliminate the potential influence of distribution extremes, we selected the CBC circles from the central 95% of the latitudinal distribution of each species’ wintering range. Based on species occurrence data from CBC circles, the northern study area boundaries ranged from 51.2 to 53.5**°**N, and southern boundaries ranged from 27.8 to 31.9**°**N.

**Table 1:** Distribution changes of six North American wintering raptor species.

Species	Num. CBCs	*β_1_*	*β_2_*	Continental Lat. CA	Western Lat. CA^a^
American Kestrel	211	0.336	−2.65×10^−4^	0.44	4.14 (3.24, 5.04)
Golden Eagle	353	0.034	−3.53×10^−5^		7.74 (6.61, 8.86)
Northern Harrier	212	0.156	−1.64×10^−4^	3.94	1.52 (0.58, 2.45)
Prairie Falcon	330	0.020	−1.96×10^−5^	1.03	3.30 (1.86, 4.72)
Red-tailed Hawk	295	0.541	−4.42×10^−4^	6.95	5.65 (4.70, 6.60)
Rough-legged Hawk	279	0.112	−3.16×10^−4^	5.94	8.41 (6.96, 9.86)

The number of Christmas Bird Count circles (Num. CBCs), coefficient values for each wintering raptor species effort corrected count (*β_1_* and *β_2_*), parameter estimates for the effect of year on continental latitude center of abundance (Continental Lat. CA; from La Sorte and Thompson [5]), and parameter estimates with 95% confidence intervals (2.5^th^ –97.5^th^ percentiles) for the effect of year on western North American latitude center of abundance for six raptor species from 1975 to 2011 using Christmas Bird Counts. The continental estimate of change in latitude center of abundance was not available for Golden Eagles.

a =  all year estimates are in km yr-1.

We selected species-specific western North American longitudinal divides based on banding and recovery data from the North American Bird Banding Program and previous studies of raptor flyways [23]. Northern Harriers and American Kestrels rarely migrated across the Rocky Mountains so we selected the continental divide as their eastern range boundary. Red-tailed Hawks and Rough-legged Hawks were generally north-south migrants, and we used the eastern edge of the Rocky Mountains as their eastern range boundary (102.0**°**W) to incorporate all three (Pacific, Intermountain, Rocky Mountain) western North American migratory flyways [23]. Golden Eagles and Prairie Falcons were generally restricted to the western United States and Great Plains region during winter [31], and we used 95.0**°**W, roughly the eastern border of Oklahoma and Kansas, as their eastern range boundary.

We calculated effort-corrected counts (*m_i_*) for each species because effort was not constant over the history of a CBC circle and observer-count relationships were likely to be species-specific [32]. We used the following effort-corrected count in our analysis:
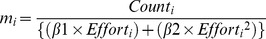



We assessed the relationship between count and effort, and found a quadratic relationship to be the best fit for all species, however the nature of this relationship varied by species. 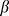
1 and 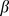
2 represent the species-specific quadratic relationship between count and effort (Table 1). We used this effort-corrected count to calculate the weighted center of latitudinal abundance following La Sorte and Thompson [5] where *m_i_* is the effort-corrected count at latitude *y_i_* for *n* CBC circles:
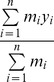



We created linear models to assess the effect of scaled year on the latitudinal center of abundance for each species to examine change in distribution over time. We performed a Breusch-Godfrey test for residual temporal autocorrelation of order up to five for all species and found no evidence for temporal autocorrelation.

### Bird Conservation Region Population Indices

We selected species-specific sets of Bird Conservation Regions (Fig. S1) within a species western wintering range (see above) for local trend analysis. BCRs with ≤100 total surveys years, corresponding to ≤3 CBC circles within the BCR, were merged with neighboring BCRs containing >100 total survey years (see Table 2, Table 3) to avoid difficulties with model convergence characteristic of small sample sizes. Although choosing to merge BCRs with ≤100 total survey years was arbitrary, we do not think this biased the results of our study. The Sierra Nevada Mountains in California and BCRs along a species range boundary were most often merged to increase sample sizes.

**Table 2:** The effect of distribution shift, year, or a full model with shift and year on northern Bird Conservation Region population indices.

Species	Models	Northern Pacific Rainforest	Sierra Nevada	Great Basin	Northern Rockies	Prairie Potholes	Badlands and Prairies	Southern Rockies/Colorado Plateau	Eastern Tallgrass Prairie
American Kestrel	Shift	**0.08 (0.01)**		**0.07 (0.01)** a	0.06 (0.04)				
	Year	−**0.16 (0.01)**		**0.23 (0.01)**	**0.09 (0.04)**			−**0.15 (0.03)**	
	Top Mod.	Full		Full	Full			Year	
Golden Eagle	Shift			0.03 (0.02)	0.04 (0.03)			−**0.05 (0.03)**	
	Year		**0.20 (0.09)**	−**0.08 (0.02)**	**0.10 (0.03)**		**0.11 (0.04)**	−0.05 (0.03)	
	Top Mod.	Intercept	Year	Full	Full	Intercept^b^	Year	Full	Intercept
Northern Harrier	Shift				**0.14 (0.05)**				
	Year		**0.19 (0.10)**	**0.09 (0.02)**	**0.20 (0.04)**			−**0.13 (0.04)**	
	Top Mod.	Intercept	Year	Year	Full			Year	
Prairie Falcon	Shift				**0.13 (0.04)**	**0.22 (0.06)**	**0.14 (0.07)**	−**0.11 (0.05)**	
	Year			−**0.04 (0.02)** a		**0.16 (0.07)**		−**0.26 (0.05)**	
	Top Mod.	Intercept		Year	Shift	Full	Shift	Full	Intercept^d^
Red-tailed Hawk	Shift	**0.05 (0.01)**		**0.04 (0.02)**			−**0.15 (0.05)** c		
	Year	**0.10 (0.01)**	**0.11 (0.05)**	**0.42 (0.02)**	**0.54 (0.03)**		**0.58 (0.05)**	**0.22 (0.02)**	
	Top Mod.	Full	Year	Full	Year		Full	Year	
Rough-legged Hawk	Shift	−0.04 (0.03)		−**0.15 (0.02)** a	**0.06 (0.03)**	**0.19 (0.06)** b		−**0.18 (0.04)**	
	Year	−**0.35 (0.03)**		−**0.22 (0.02)**	**0.22 (0.03)**	**0.41 (0.06)**	**0.47 (0.04)**	−**0.64 (0.12)**	
	Top Mod.	Full		Full	Full	Full	Year	Full	

Parameter estimates and standard errors (in parentheses) for separate models including the effect of residual latitudinal center of abundance (Shift), year (Year), or both (Full) explaining Christmas Bird Count population indices within Bird Conservation Regions for western North American raptors. Parameter estimates are from the top ranked model (Top Mod.; see Table S1 for delta AIC values). Bolded numbers indicate 85% confidence intervals that do not overlap zero. Blank cells indicate Bird Conservation Regions outside of the wintering range analyzed for each species in this study. See footnotes for combined Bird Conservation Regions because of insufficient samples sizes.

a =  Sierra Nevada merged with Great Basin;

b =  Boreal Taiga Plains merged with Prairie Potholes;

c =  Boreal Taiga Plains and Prairie Potholes merged with Badlands and Prairies;

d =  Prairie Hardwood Transition merged with Eastern Tallgrass Prairie.

**Table 3:** The effect of distribution shift, year, or a full model with shift and year on southern Bird Conservation Region population indices.

Species	Models	Coastal California	Sonoran andMohave Deserts	Sierra MadreOccidental	ChihuahuanDesert	ShortgrassPrairie	Central Mixed-grass Prairie	Oaks and Prairies	Gulf Coast Prairie
American Kestrel	Shift	−**0.05 (0.01)**		−**0.05 (0.03)**					
	Year	−**0.23 (0.01)**		−**0.09 (0.03)**					
	Top Mod.	Full	Intercept	Full					
Golden Eagle	Shift	−**0.03 (0.02)**	−0.08 (0.06)	−0.07 (0.04)	−**0.09 (0.04)**				
	Year	−**0.07 (0.02)**	−**0.15 (0.06)**	−**0.14 (0.04)**	−**0.15 (0.05)**	−**0.06 (0.03)**	−**0.62 (0.10)**	−**0.32 (0.13)** a	
	Top Mod.	Full	Full	Full	Full	Year	Year	Year	Intercept
Northern Harrier	Shift	−**0.10 (0.01)**	−**0.08 (0.03)**						
	Year								
	Top Mod.	Shift	Shift	Intercept					
Prairie Falcon	Shift	−**0.06 (0.02)**	−**0.08 (0.04)**	−**0.13 (0.05)**		−**0.05 (0.03)**			
	Year	−**0.07 (0.02)**		−**0.24 (0.05)**			**0.17 (0.06)**		
	Top Mod.	Full	Shift	Full	Intercept	Shift	Year	Intercept^a^	
Red-tailed Hawk	Shift	−**0.01 (0.01)**			−**0.06 (0.03)**				
	Year	**0.03 (0.01)**	**0.10 (0.02)**	**0.07 (0.02)**	**0.08 (0.03)**	**0.44 (0.02)**			
	Top Mod.	Full	Year	Year	Full	Year			
Rough-legged Hawk	Shift	−**0.16 (0.03)**	−**0.18 (0.10)**	−**0.32 (0.10)**		−**0.13 (0.03)**			
	Year	−**0.40 (0.04)**	−**0.64 (0.12)**	−**0.37 (0.12)**	−**0.39 (0.09)**	−**0.34 (0.03)**			
	Top Mod.	Full	Full	Full	Year	Full			

Parameter estimates and standard errors (in parentheses) for separate models including the effect of residual latitudinal center of abundance (Shift), year (Year), or both (Full) explaining Christmas Bird Count population indices within Bird Conservation Regions for western North American raptors. Parameter estimates are from the top ranked model (Top Mod.; see Table S1 for delta AIC values). Bolded numbers indicate 85% confidence intervals that do not overlap zero. Blank cells indicate Bird Conservation Regions outside of the wintering range analyzed for each species in this study. See footnotes for combined Bird Conservation Regions because of insufficient samples sizes.

a =  Edwards Plateau, West Gulf Coastal Plain/Quachitas, and Tamaulipan Brushlands merged with Oaks and Prairies.

We created generalized linear mixed models with a negative-binomial distribution to examine evidence for the effect of year, residual distribution, or both on annual CBC counts within each BCR. We used the residuals from a linear model of latitude center of abundance and year to represent distribution shift not explainable by year because year and latitude center of abundance were highly collinear (r = 0.50 to 0.90). Predictors were scaled and centered to allow for direct comparisons of parameter estimates. We included observer effort as an “offset” term in the model. Circle ID was used as a random effect in all BCR models. We fit models using the ‘nbinom’ family corrected for overdispersion (zeroInflation = TRUE) within the glmmADMB package in R-statistical (2013) and used an information-theoretic approach with Akaike’s Information Criterion (AIC; [33]) to evaluate model support. Specifically, we examined whether local BCR populations were best explained by year, residual latitude center of abundance, or both by comparing AIC values [33] from competing models. Models with the lowest AIC were considered the model with the most support, and we calculated 85% confidence intervals for parameter estimates to be compatible with an AIC approach [34]. In some cases, other models had a delta AIC of <2 but broad confidence intervals around parameter estimates in second ranked models suggested unreliable parameter estimates; we therefore reported only the top model and associated parameter and standard error estimates. We checked for residual spatial autocorrelation by creating a spline correlogram [35] of the Pearson residuals from BCR- and species-specific models for the effect of year or residual annual latitudinal center of abundance on counts. We found no evidence for residual spatial autocorrelation in our models. We also checked for residual temporal autocorrelation in our BCR analyses by plotting the Pearson residuals from BCR- and species-specific models against year and again found no evidence for residual temporal autocorrelation.

For overall western winter population trend analyses we created linear models for the effect of year on the mean annual effort-corrected count for each species. We assessed the linear fit of year on mean annual effort-corrected count for each species to determine any quadratic trends in population change over time. All statistical analyses were run with software from the R Development Core Team (2013).

## Results

**Figure 1:**
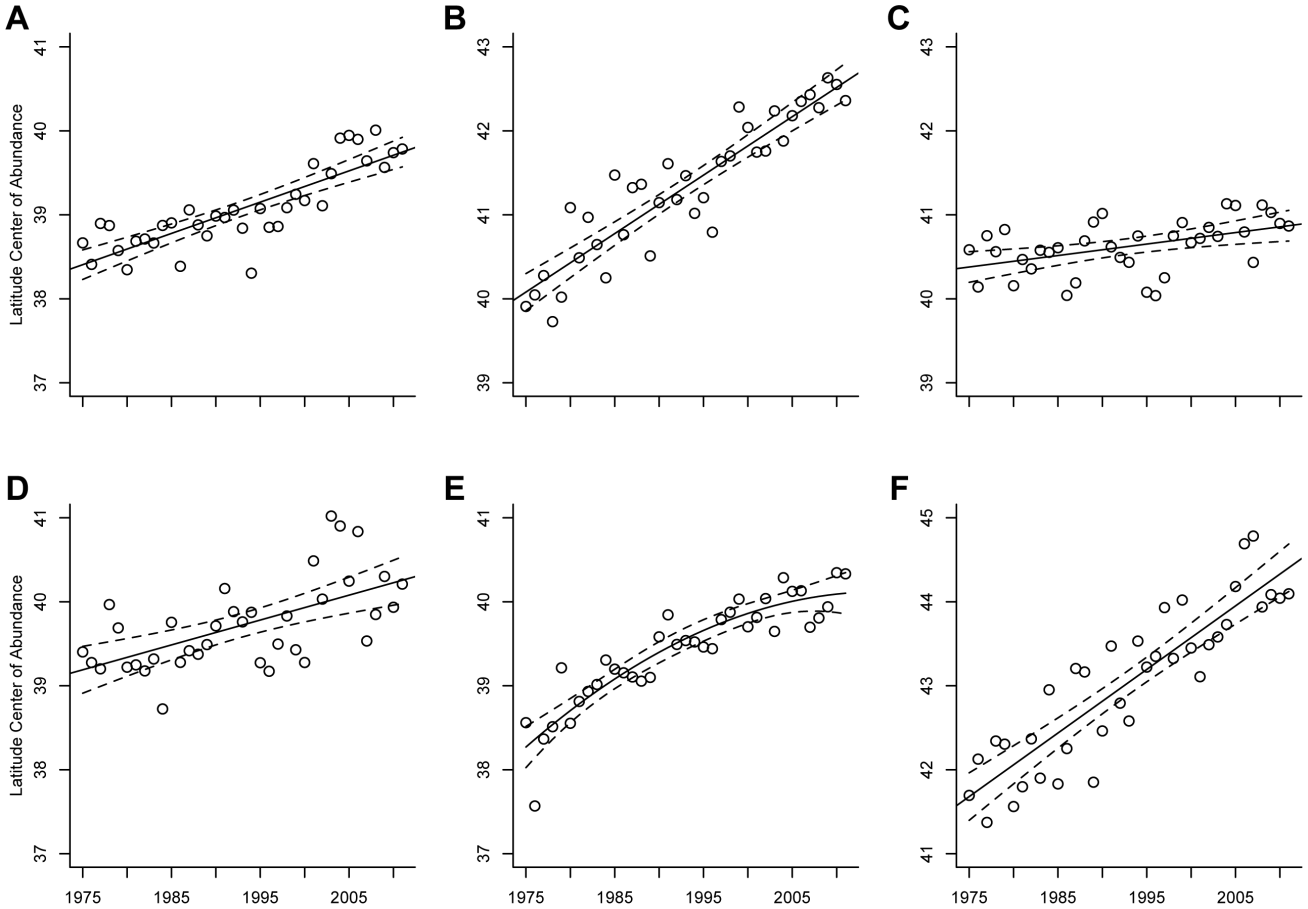
Relationship between year and distribution for six wintering raptor species. The relationship between year and latitude center of abundance (° latitude) for (**A**) American Kestrels, (**B**) Golden Eagles, (**C**) Northern Harriers, (**D**) Prairie Falcons, (**E**) Red-tailed Hawks, and (**F**) Rough-legged Hawks in western North American Christmas Bird Counts from 1975 to 2011. Solid lines indicate a predictive relationship and dashed lines represent 95% confidence intervals.

All species showed evidence for a northward shift in their latitudinal center of abundance over time (Fig. 1; American Kestrels: t = 8.99, df = 35, *P*<0.0001; Golden Eagles: t = 12.44, df = 35, *P*<0.0001; Northern Harriers: t = 3.18, df = 35, *P* = 0.003; Prairie Falcons: t = 4.51, df = 35, *P*<0.0001; Red-tailed Hawks: t = 11.70, df = 35, *P*<0.0001; Rough-legged Hawks: t = 11.37, df = 35, *P*<0.0001). In general, the degree of northward shift we observed differed from continental estimates ([5], Table 1).

One explanation for an apparent northern shift in distribution may be that the locations of CBC circles have shifted north over time. However, because we only included long-term circles surveyed in at least 9 of 12, 3-year time periods we did not find significant correlations between year and latitude of CBC circles (Northern Harriers and American Kestrels: *r* = 0.01, df = 7,142, *P* = 0.26; Rough-legged Hawks: *r* = 0.02, df = 9,847, *P* = 0.11; Red-tailed Hawks: *r* = 0.02, df = 9,513, *P* = 0.12; Golden Eagles: *r* = 0.01, df = 13,628, *P* = 0.36; Prairie Falcons: *r* = 0.01, df = 13,499, *P* = 0.36).

Relationships between BCR population indices and residual distributions or time (year) depended on species and BCR. There was greater evidence that the residual latitudinal center of abundance not explained by year improved BCR population index model fit in American Kestrels, Prairie Falcons, Red-tailed Hawks, and Rough-legged Hawks (Table 2, Table 3). Alternatively, there was less evidence that the residual latitudinal center of abundance improved model fit for Northern Harriers and Golden Eagles (Table 2, Table 3). Across all species, model fits were improved for 40% of species-specific BCR population indices when species distribution information was included in the form of residual latitude center of abundance (Table S1).

**Figure 2:**
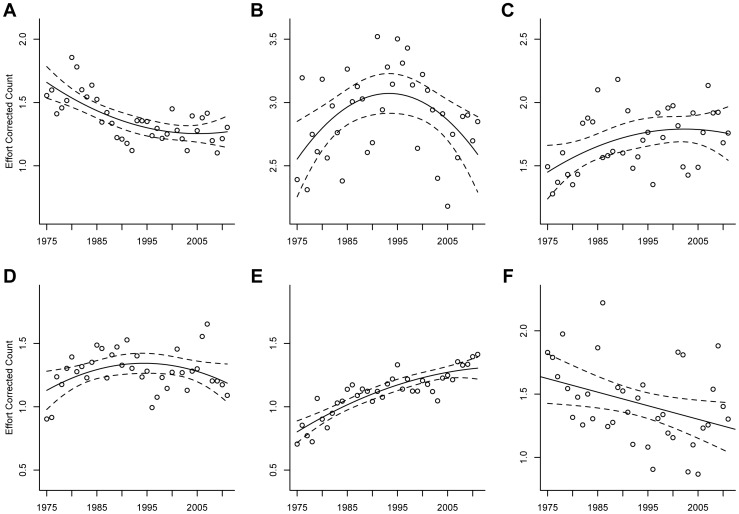
Western North America population trends for six wintering raptor species. The relationship between year and mean effort-corrected count (raptors/(hours+hours^2^)) for (**A**) American Kestrels, (**B**) Golden Eagles, (**C**) Northern Harriers, (**D**) Prairie Falcons, (**E**) Red-tailed Hawks, and (**F**) Rough-legged Hawks in western North American Christmas Bird Counts from 1975 to 2011. Solid lines indicate a predictive relationship and dashed lines represent 95% confidence intervals.

For all species except Rough-legged Hawks a quadratic trend estimating overall winter population trends from 1975 to 2011 provided the best model fit (Fig. 2). These include negative quadratic relationships for Golden Eagles, Northern Harriers, Prairie Falcons, and Red-tailed Hawks and a positive quadratic relationship for American Kestrels. Rough-legged Hawks showed a negative linear overall population trend (Fig. 2F); however, this may have been caused by a lack of adequate sampling of northern populations as we found evidence for a negative relationship between the yearly latitudinal center of abundance and yearly average abundance (Fig. S2).

## Discussion

All six raptor species showed a northward shift in their western North American winter distributions. Changes in distribution explained a high proportion of variation in annual CBC counts and improved interpretation of local Bird Conservation Region (BCR) population indices. These results indicate that natural resource managers must consider the influence of shifting distributions when drawing inferences about drivers of trends in local populations.

Northward shifts in wintering distribution are consistent with results from continental studies of birds in North America [5]. However, the estimates for the rate of shift in western regions differed from continental estimates. For species that we were able to compare with La Sorte and Thompson’s [5] continental analysis, we found higher distribution shifts for American Kestrels, Prairie Falcons, and Rough-legged Hawks and lower shifts for Northern Harriers and Red-tailed Hawks. We were unable to compare Golden Eagle distribution shifts because La Sorte and Thompson [5] did not analyze this species. Our results highlight the need to study distribution changes at a variety of scales, and for a variety of species. Specifically, regional distribution estimates may more accurately reflect rates of change in local populations compared to continent-wide estimates that may be influenced by inclusion of populations experiencing different changes in climate or with different life history characteristics [27].

Several studies have found decreased avian migration distances and northward shifts in distributions to be influenced by climate change [15], [21]. Decreased migration distances in American Kestrels from western North America are strongly associated with warming temperatures [21]. Decreased migration distances, migratory “short-stopping”, and increased winter residency are all possible explanations for our observed distribution shifts [36]. If raptors decrease their migration distances and winter further north or stay on breeding grounds through the winter, they are at an advantage because early arrival to the breeding grounds may positively predict territory quality and increase reproductive success in species such as Prairie Falcons [31], Merlins (*Falco columbarius*; [22]) and American Kestrels [37].

An alternative explanation for an apparent northern shift in wintering distributions is differential land-use change in southern areas contributing to habitat destruction and degradation, which effectively “pushes” raptors further north to areas with higher habitat suitability [38]. While it was beyond the scope of this work, anthropogenic factors such as increasing human populations and development should be assessed in future range shift studies [38]. Other variables not assessed in this study that could influence raptor distribution include differential habitat loss, alteration of prey distribution and abundance, temperature, snow cover, precipitation, other weather variables, and raptor population life-history variability [39], [38].

Rough-legged Hawks had the largest observed northward distribution shift (8.41 km yr^−1^ over the last 36 years). Considering that temperature may influence migratory distance [40], we would expect to see pan-arctic species potentially most affected by temperature changes [27] and exhibit more pronounced distribution shifts. Also, Rough-legged Hawks have extensive arctic breeding populations and provided the clearest support for distribution shifts explaining BCR population indices suggesting some long-distance arctic migrants may be more likely to experience distribution shifts that influence local population indices. Approximately 64% (7 of 11) of Rough-legged Hawk BCR population indices were better explained by including information on distribution shifts.

We found evidence that distribution shifts explained variation in several BCR population indices and contributed information about changes in population size; although this varied by species and by geographic location of BCRs. In general, CBC counts in northern BCRs such as the Northern Rockies increased with distribution shifts to the north and CBC counts in southern BCRs decreased. Coastal California and northern areas, including the Prairie Potholes and Northern Rockies BCRs, provided the most evidence for distribution shifts improving our understanding of BCR population indices. Other BCRs provided mixed evidence for distribution shifts improving our understanding of population indices. The Southern Rockies/Colorado Plateau and Great Plains BCRs provided evidence for population changes less likely to be attributable to distribution shifts.

Models of local population change that do not take into account the effect of regional distribution shifts may be misinterpreted by researchers and land managers. Frequently, parameter estimates for distribution shifts and year had the same direction of effect. This suggests that some trends of increasing populations in northern BCRs were partly explained by regional distribution shifts, and trends of declining populations in southern BCRs may be partly explained by distribution shifts. In BCRs where the effect of distribution was opposite that of year, regional distribution shifts may create ambiguous trend analyses. For example, CBC counts of American Kestrels in the Northern Pacific Rainforest BCR were positively associated with northward shifts, but negatively associated with year suggesting a decline in local wintering populations. This decline may be offset by a population increase attributable to regional changes in wintering distribution. Alternatively, if climate change is contributing to regional distribution shifts at southern sites, local population declines might erroneously be attributed to land uses changes that lead to decreased habitat suitability. For example, Prairie Falcons and Rough-legged Hawks showed declining population indices in some southern BCRs but these may be largely attributable to regional distribution changes. If the wintering distribution of a species is shifting poleward, habitat restoration or improvement efforts at southern sites may have little effect on target species because of improving climatic conditions to the north.

In BCRs experiencing declines less likely to be influenced by distribution shifts, additional research should focus on determining the causes of winter population declines and how they may be mitigated. For example, American Kestrels and Northern Harriers had declining population trends in the Southern Rockies/Colorado Plateau BCR, but there was little evidence that these trends were explained by changes in latitudinal distribution over time. Most concerning was evidence that several declining BCR population trends for Golden Eagles were not likely to be attributable to distribution shifts. For Golden Eagles, negative population trends were found in eight BCRs, with distribution shift only explaining CBC counts in two BCRs. Consequently, we believe it is critically important to continue monitoring Golden Eagle populations and movement patterns. Data on breeding populations could provide direct evidence that changes not directly related to distribution shifts explain BCR population indices. Unfortunately, data on potential causes of declines in breeding populations can be difficult to obtain for species whose breeding ranges extend into Canada and the high arctic, where fewer long-term breeding surveys are conducted [41]. Another difficulty in connecting breeding population changes to wintering distributions and population trends is assigning connectivity between wintering and breeding areas. We did not attempt to explain changes in wintering distributions and populations from data on breeding raptors because of these difficulties.

An apparent decline in overall population size could result from some northern wintering populations not being effectively sampled in our analysis, or by a lack of adequate sampling in extreme northern locations by CBC circles. If winter distributions have shifted so far north that they are now outside of the CBC sampling area, CBC data would indicate a decline in wintering populations. We saw evidence for this in only one of the six-raptor species: Rough-legged Hawks. This suggests that the apparent western North American population decline in Rough-legged Hawks may be because of an inadequately sampled northern wintering population. Perhaps this area is not adequately sampled by the CBC or was eliminated by our experimental methodology. However, we know of no other study that has assessed Rough-legged Hawk populations directly. Moreover, little to no research has been conducted on Rough-legged Hawk breeding populations in the past two decades [42]. Such studies would be useful to test our hypothesis of a stable Rough-legged Hawk wintering population moving northward in western North America. Rough-legged Hawks may be a model species for how climate change can impact distributions, populations, and movements given their extensive arctic breeding range, and the large winter distribution shift documented in this study.

Future studies and analyses of wintering raptors in western North America should attempt to explore how human population growth and human activities are influencing distribution shifts and BCR population trends. Teasing apart the effects of climate, human populations, and distribution shifts may be difficult as human population growth tends to be highest in warmer, southern areas such as California and Arizona that exhibit declining raptor population trends. As the global climate continues to warm, species wintering ranges may continue to shift further north. As a consequence, natural resource managers in northern areas may become responsible for managing an increasing proportion of a species wintering population. Such changes are likely to prompt agencies with the mission of managing such species to reallocate their resources. This will be critical for population persistence in the future given the importance of winter survival on avian population demography.

## Supporting Information

Figure S1 **Western North American Bird Conservation Regions.** Map of all of the Bird Conservation Regions (BCRs) included in our analysis of six western North American raptor species.
(TIFF)

Figure S2 **Relationship between distribution and annual count for wintering Rough-legged Hawks.** The relationship between the latitudinal center of abundance (° latitude) and average yearly effort-corrected abundance (raptors/(hours+hours^2^)) for Rough-legged Hawks in western North American Christmas Bird Counts from 1975 to 2011. Presence of a line indicates a predictive relationship.
(TIF)

Table S1 **The effect of distribution shift and/or year on Bird Conservation Region population indices.** Delta AIC values and number of parameters (k) in parentheses for models including the effect of residual latitudinal center of abundance (Shift), year (Year), or both (Full) explaining Christmas Bird Count population indices within Bird Conservation Regions for western North American raptors. Blank cells indicate Bird Conservation Regions outside of the wintering range analyzed for each species in this study. See Table 2 footnotes for combined Bird Conservation Regions because of insufficient samples sizes.
(XLSX)
