# Supplementary figures and images for: Regional Distribution Shifts Help Explain Local Changes in Wintering Raptor Abundance: Implications for Interpreting Population Trends
Source: PLoS One. 2014 Jan 22;9(1):e86814. doi: 10.1371/journal.pone.0086814 (PMC3899332; doi:10.1371/journal.pone.0086814)

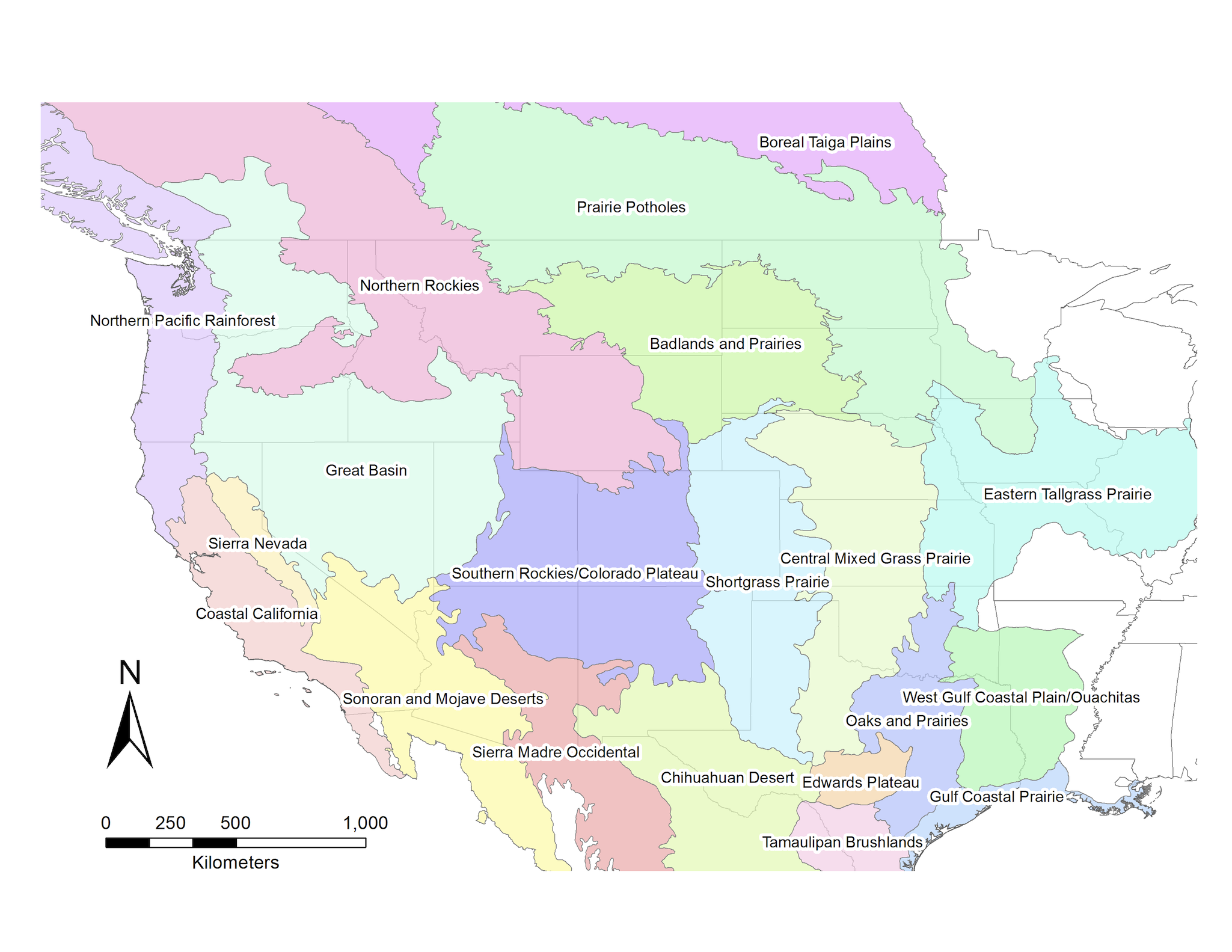

Supplement: Figure S1 — Western North American Bird Conservation Regions. Map of all of the Bird Conservation Regions (BCRs) included in our analysis of six western North American raptor species. (TIFF) [file pone.0086814.s001.tiff]

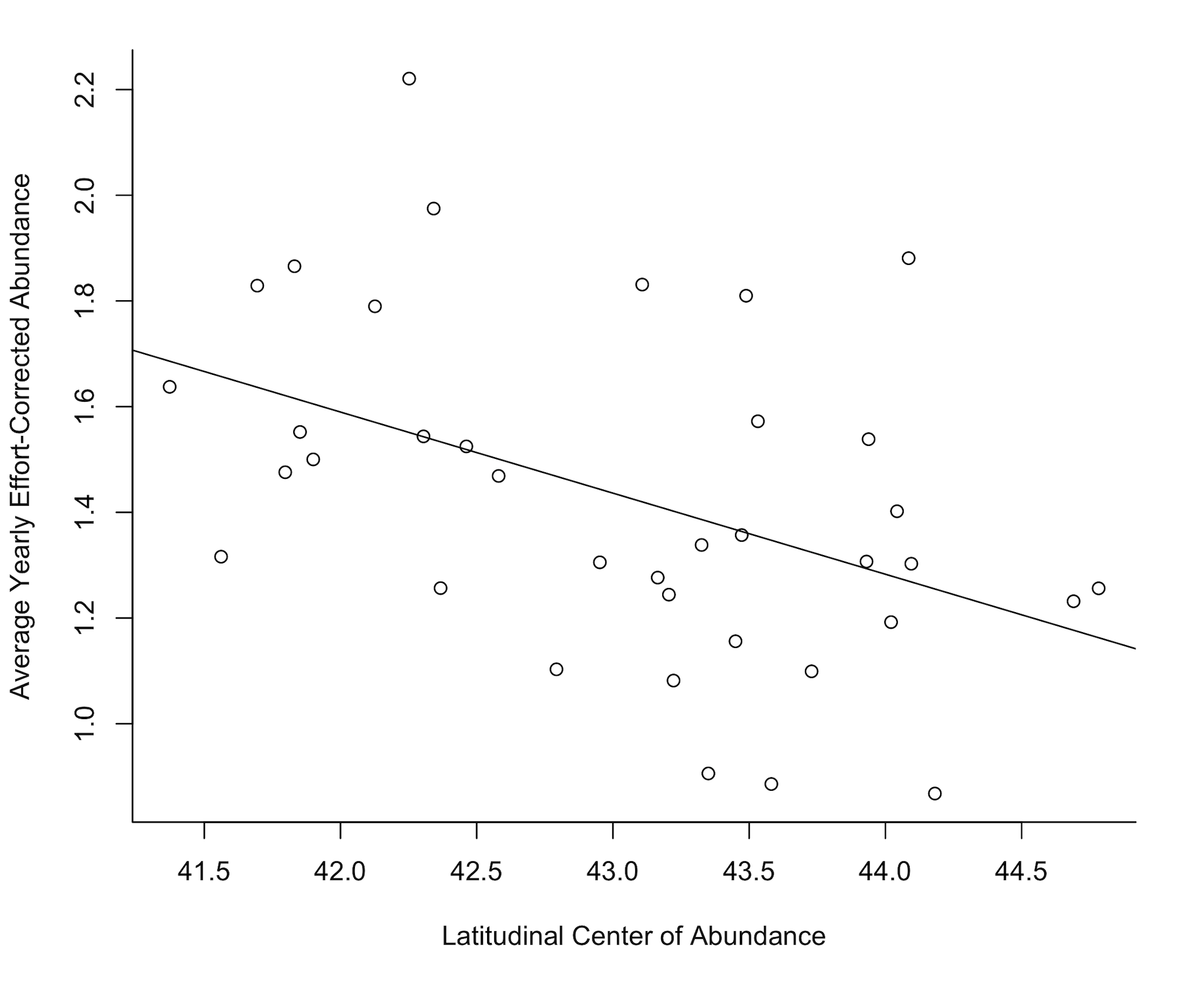

Supplement: Figure S2 — Relationship between distribution and annual count for wintering Rough-legged Hawks. The relationship between the latitudinal center of abundance (° latitude) and average yearly effort-corrected abundance (raptors/(hours+hours2)) for Rough-legged Hawks in western North American Christmas Bird Counts from 1975 to 2011. Presence of a line indicates a predictive relationship. (TIF) [file pone.0086814.s002.tif]
